# MicroRNA‐150 serves as a diagnostic biomarker and is involved in the inflammatory pathogenesis of Parkinson's disease

**DOI:** 10.1002/mgg3.1189

**Published:** 2020-02-20

**Authors:** Haiting Li, Ling Yu, Min Li, Xiaohui Chen, Qun Tian, Yanyan Jiang, Nan Li

**Affiliations:** ^1^ Department of Neurology Shengli Oilfield Central Hospital Dongying Shandong China

**Keywords:** AKT signaling, diagnosis, MicroRNA‐150, neuroinflammation, Parkinson's disease

## Abstract

**Background:**

Dysregulation of microRNAs (miRNAs) has been reported to be involved in the neuroinflammatory pathogenesis of PD. This study aimed to investigate the serum expression of microRNA‐150 (miR‐150) in Parkinson's disease (PD) patients and further uncover the regulatory effect of miR‐150 on neuroinflammation.

**Methods:**

Quantitative Real‐Time PCR was used to measure the expression of miR‐150. A receiver operating characteristic curve was applied to evaluate the diagnostic value of miR‐150. The effect of miR‐150 on neuroinflammation was analyzed by examining its correlation with proinflammatory cytokines and gain‐of‐function experiments in microglia treated with LPS.

**Results:**

Serum miR‐150 expression was downregulated in PD patients compared with the healthy controls, and served as a candidate diagnostic biomarker for the screening of PD cases. Negative correlation was found between miR‐150 levels and the levels of procytokines in PD patients. By the treatment of LPS, microglia BV2 cells had a reduced expression of miR‐150, and the enhanced neuroinflammatory responses were inhibited by the overexpression of miR‐150. AKT3 was verified as a target of miR‐150 in BV2 cells.

**Conclusion:**

All the data of this study revealed that the decreased serum miR‐150 serves as a potential diagnostic biomarker. The methods to increase miR‐150 expression may have a beneficial effect in PD via suppressing the neuroinflammation by targeting AKT3.

## Graphical abstract

The downregulated expression of miR‐150 serves as a diagnostic biomarker of Parkinson's disease.Serum miR‐150 levels are correlated with Parkinson's patients' inflammation and miR‐150 is decreased in microglia by LPS treatment. miR‐150 can inhibit neuroinflamation by targeting AKT3.

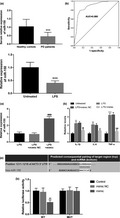

## 1 INTRODUCTION

Parkinson's disease (PD) is a frequent neurodegenerative disease with the incidence rate of 2% among the population over 65 years old (Ascherio & Schwarzschild, 2016). PD is pathologically characterized by the selective loss of dopaminergic neurons in the midbrain substantia nigra pars compacta and the presence of intracytoplasmic protein aggregates (Liu et al., 2016). Patients with PD have some motor and nonmotor symptoms, such as rigidity, resting tremor, bradykinesia, postural instability, and akinesia for the motor symptoms and cognitive dysfunction, sleep disorder, psychiatric symptoms, autonomic dysfunction, pain, olfactory dysfunction, and fatigue for the nonmotor manifestations (Kalia & Lang, 2015). The diagnosis of PD is mainly dependent on the clinical symptoms and neuroimaging, which is only suitable for the patients who appear motor features (Massano & Bhatia, 2012). Thus, histopathology is considered the “gold standard” for the diagnosis of PD, but its application is severely limited by the invasiveness of the procedure and the incorrect sampling that may have occurred in random brain biopsy (Reschke & Henshall, 2015; Sharma et al., 2013). Therefore, non‐invasive and efficient diagnostic methods are urgently needed to improve the diagnosis of PD.

Currently, great efforts have been carried out for the treatment of PD, but the strategies that could significantly improve PD clinical features remain limited (Kalia, Kalia, & Lang, 2015). The pathogenesis of PD is complex, the understanding about it, however, is still not enough for the exploration of effective therapeutic methods (Alexoudi, Alexoudi, & Gatzonis, 2018). Neuroinflammation plays a critical role in the progression of PD (Poewe et al., 2017). Microglia, as the major immune cells of the central nervous system, have been demonstrated to be activated in the lipopolysaccharide (LPS)‐induced PD models (Huang et al., 2017; More & Choi, 2017). The activation of microglia mediates inflammatory responses in brain and leads to the release of proinflammatory cytokines, such as interleukin (IL)‐1β, IL‐6, and tumor necrotic factor (TNF)‐α, which contributes to the development of PD (Schwenkgrub et al., (2017)). The pivotal role of neuroinflammation suggests that the methods to inhibit neuroinflammation may be novel therapeutic strategies for the treatment of PD.

MicroRNAs (miRNAs) are a group of small noncoding RNAs with important regulatory function in various processes under normal and disease conditions (Vienberg, Geiger, Madsen, & Dalgaard, 2017). Aberrant expression of miRNAs in disease progression and its stability in body fluid, especially in serum, leading to miRNAs becomes a class of biomarkers for the diagnosis of various diseases (Piscopo et al., 2018; Qiu, Li, Wang, & Sun, 2017). In PD patients, some miRNAs with abnormal expression patterns have been identified as candidate diagnostic biomarkers, such as miR‐29 (Bai et al., 2017) and miR‐221 (Ma et al., 2016). In addition to the clinical significance, the therapeutic potentials of miRNAs have also been highlighted through regulating neuroinflammation in PD progression (Cao, Wang, Qu, Kang, & Yang, 2018). Similar to PD, Alzheimer's disease also is a common neurodegenerative disease. Lugli et al. have found that the expression of microRNA‐150 (miR‐150) was significantly decreased in Alzheimer's disease (Lugli et al., 2015). In addition, the regulatory effect of miR‐150 on neuroinflammatory responses has been reported in neuropathic pain (Ji, Shi, Lu, & Huang, 2018). However, the expression and biological function in the progression of PD remain unclear.

To explore novel molecule that has diagnostic and therapeutic potential in PD progression, this study aimed to investigate the serum expression of miR‐150 in patients with PD and further explore the relationship of miR‐150 with neuroinflammation in patients and microglia.

## 2 MATERIALS AND METHODS

### 2.1 Patients and sample collection

A total of 80 PD patients were enrolled from the Shengli Oilfield Central Hospital between 2015 and 2017, and a control group, including 60 age‐ and gender‐matched healthy volunteers, was recruited in this study. The diagnosis of PD was performed according to the UK PD Brain Bank criteria (Joutsa, Gardberg, Roytta, & Kaasinen, 2014). The experimental protocols were approved by the Ethics Committee of the Shengli Oilfield Central Hospital, and an informed consent was obtained from each participant. Blood samples were collected from the participants and immediately centrifuged for the isolation of serum. The collected serum samples were stored at −80℃ for further uses. In addition, the demographic and clinicopathological features of the patients were record for subsequent analyses.

### 2.2 Cell culture and treatment

Microglial cells BV2 were purchased from the Cell Bank of Chinese Academy of Medical Science (Shanghai, China) and cultured in Dulbecco's modified Eagle's medium (DMEM; Gibco) containing 10% fetal bovine serum (FBS; Invitrogen) at 37℃ in a humidified atmosphere with 5% CO_2_. To simulate the neuroinflammation in PD progression, BV2 cells were exposed to 1 µg/ml of LPS for 12 hr. miR‐150 mimic (GenePharma) was transfected into the BV2 cells to upregulate the expression of miR‐150 in vitro. The mimic negative control (mimic NC; GenePharma) was transfected into the BV2 cells to serve as a control. The sequences of vectors were as follows: miR‐150 mimic 5'‐UCUCCCAACCCUUGUACCAGUG‐3' and mimic NC 5'‐UUCUCCGAACGUGUCACGU‐3'. Cell transfection was performed using the Lipofectamine 3000 (Invitrogen) following the manufacturer's instruction. The subsequent cell experiments were conducted at 48 hr after cell transfection.

### 2.3 RNA extraction

Total RNA in the collected serum samples and BV2 cells was extracted using TRIzol reagent (Invitrogen) according to the manufacturer's instruction. The concentration and purity of the RNA were checked using a NanoDrop 2000 (Thermo Fisher Scientific). One µg of total RNA was reversely transcribed into cDNA by a PrimeScript RT reagent kit (TaKaRa) following the protocol of manufacture.

### 2.4 Quantitative real‐time PCR (qRT‐PCR)

The synthesized cDNA was used as a template of qRT‐PCR, which was performed using a SYBR green I Master Mix kit (Invitrogen) on a 7500 Real‐Time PCE System (Applied Biosystems). U6 was used as an internal control in the reactions, and the reaction condition was as follow: 95℃ for 10 min, 95℃ for 30 s, 60℃ for 20 s, 72℃ for 15 s, a total of 35 cycles. Following are the sequences of primers: miR‐150 forward: 5′‐GCCGAGTCTCCCAACCCTT‐3′, reverse: 5′‐CTCAACTGGTGTCGTGGA‐3′; U6 forward: 5′‐CTCGCTTCGGCAGCACA‐3′, reverse: 5′‐AACGCTTCACGAATTTGCGT‐3′. The relative expression of miR‐150 was calculated using the 2^−ΔΔCt^ method and normalized to U6.

### 2.5 Enzyme‐linked immunosorbent assay (ELISA)

To evaluate the inflammatory responses, levels of IL‐1β, IL‐6, and TNF‐α in the serum samples and BV2 cell supernatants were measured using the ELISA kit (BioSource International, USA) following the manufacturer's instruction.

### 2.6 Luciferase activity assay

By a prediction using TargetScan (http://www.targetscan.org/vert_72/), target genes of miR‐150 were predicted. At the 3′‐untranlated region (UTR) of v‐akt murine thymoma viral oncogene homolog 3 (AKT3), the complementary sequence of miR‐150 was found. Thus, a luciferase activity assay was performed by cotransfecting the wile‐type (WT) or mutant type (MUT) 3′‐UTR of AKT3 with mimic NC or miR‐150 mimic using Lipofectamine 3000 (Invitrogen). The relative luciferase activity of BV2 cells was estimated using a Dual‐Luciferase Assay kit (Promega) according to the manufacturer's protocol.

### 2.7 Statistical analysis

Data were expressed as mean ± *SD* and analyzed using SPSS 21.0 (SPSS Inc.) and GraphPad Prism 7.0 software (GraphPad Software, Inc.). The difference between groups was analyzed using Student's *t* test, one‐way ANOVA. A receiver operating characteristic curve (ROC) was plotted to evaluate the diagnostic value of miR‐150. The correlation analysis between independent indicators was performed using a Pearson correlation analysis. A *p* value of less than .05 was considered statistically significant.

## 3 RESULTS

### 3.1 Baseline characteristics of the research cohort

The demographic characteristics of the participants, including age and gender, and the clinical information of the PD patients, including disease duration and Hoehn‐Yahr (H & Y) stage, were listed in Table 1. In comparison of the healthy control, there was no statistical significance in the age and gender between healthy controls and PD patients (both *p* > .05). The disease duration and H & Y stage of the PD patients were 4.8 ± 3.23 and 2.2 ± 0.81, respectively.

**Table 1:** Baseline characteristics of the PD patients and healthy controls

Characteristics	Healthy controls (*n* = 60)	PD patients (*n* = 80)	*p* value
Age (years, mean ± *SD*)	64.0 ± 7.29	64.6 ± 7.54	.643
Gender (male/female)	31/29	42/38	.922
Disease duration (years, mean ± *SD*)	—	4.8 ± 3.23	–
H & Y stage (mean ± *SD*)	—	2.2 ± 0.81	–

Abbreviations: H & Y, Hoehn‐Yahr; PD, Parkinson's disease.

### 3.2 Downregulated serum expression of miR‐150 in PD patients

**Figure 1:**
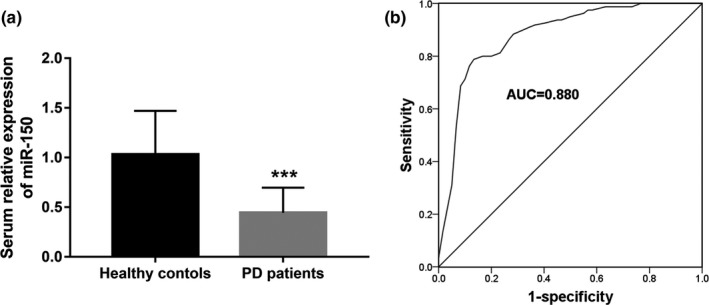
Serum expression of miR‐150 and its diagnostic value in patients with PD. (a) The expression of miR‐150 in the serum samples of PD patients was decreased compared with the healthy controls. (b) A ROC curve constructed based on the serum levels of miR‐150 for PD patients. Area under the curve (AUC) was 0.880; ***p < .001

By qRT‐PCR, the serum expression levels of miR‐150 in the patients with PD and the healthy controls were estimated. As shown in Figure 1a, a significant downregulation in the expression of miR‐150 was observed in patients with PD compared with the healthy individuals (*p* < .001).

### 3.3 Diagnostic accuracy of serum miR‐150 in patients with PD

Considering the decreased serum expression of miR‐150 in PD patients, a ROC curve was drawn to evaluate its diagnostic value. The area under the curve (AUC) shown in Figure 1b was 0.880, indicating the high diagnostic accuracy for miR‐150 to screen PD patients from the healthy controls. At an optimal cutoff value of 0.550, serum miR‐150 could be used to distinguish PD patients from healthy individuals with relatively high sensitivity (77.5%) and specificity (88.3%).

### 3.4 Correlation between miR‐150 and inflammatory response in PD patients

The serum concentration of proinflammatory cytokines was estimated in the patients with PD, and the correlation of these cytokines with serum levels of miR‐150 was further analyzed. The data shown in Table 2 indicated that serum expression levels of miR‐150 were negatively correlated with IL‐1β (*r* = −0.509, *p* < .001), IL‐6 (*r* = −0.545, *p* < .001), and TNF‐α (*r* = −0.652, *p* < .001), suggesting the potential relationship between miR‐150 and neuroinflammation of PD.

**Table 2:** Correlation of miR‐150 with levels of proinflammatory cytokines in patients with PD

Indicators	Serum relative levels	Relative miR‐150 levels	
		*r* value	*p* value
IL‐1β	1.93 ± 0.51	−.509	<.001
IL‐6	1.83 ± 0.53	−.545	<.001
TNF‐α	2.76 ± 0.66	−.652	<.001

Abbreviations: IL, interleukin; PD, Parkinson's disease; TNF, tumor necrotic factor.

### 3.5 LPS suppresses the expression of miR‐150 in BV2 cells

**Figure 2:**
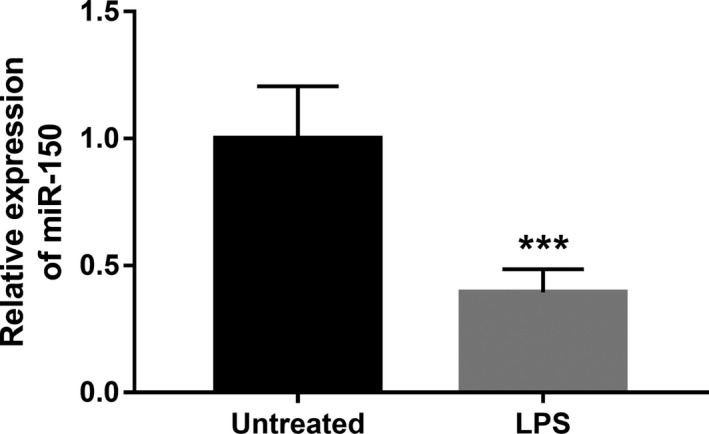
Downregulated expression of miR‐150 in BV2 cells treated with LPS. ***p < .001

Microglia BV2 cells were applied to perform the neuroinflammation analysis in vitro by the stimulation of LPS. After the exposure of LPS, the expression of miR‐150 was significantly inhibited by LPS in BV2 cells compared with the untreated cells (*p* < .001, Figure 2).

### 3.6 Overexpression of miR‐150 attenuates neuroinflammation induced by LPS treatment

**Figure 3:**
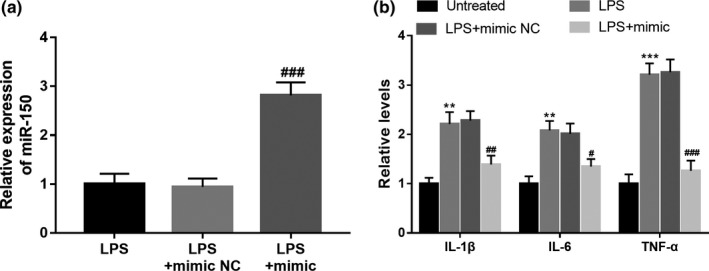
Effect of miR‐150 on neuroinflammation in microglia. (a) Expression of miR‐150 in BV2 cells was upregulated by the miR‐150 mimic. (b) The enhanced release of IL‐1β, IL‐6, and TNF‐α was significantly inhibited by the overexpression of miR‐150. **p < .01, ***p < .001 versus Untreated; ^#^ *p* < .05, ^##^ *p* < .01, ^###^ *p* < .001 versus LPS

To further confirm the effect of miR‐150 on the neuroinflammatory response in PD progression, the expression of miR‐150 in BV2 cells was regulated by cell transfection. As shown in Figure 3a, the reduced expression of miR‐150 induced by LPS was obviously elevated by the miR‐150 mimic in BV2 cells (*p* < .001). Furthermore, the enhanced release of IL‐1β, IL‐6, and TNF‐α in the BV2 cells treated with LPS was remarkedly suppressed by the overexpression of miR‐150 (all *p* < .05, Figure 3b).

### 3.7 AKT3 serves as a direct target of miR‐150

**Figure 4:**
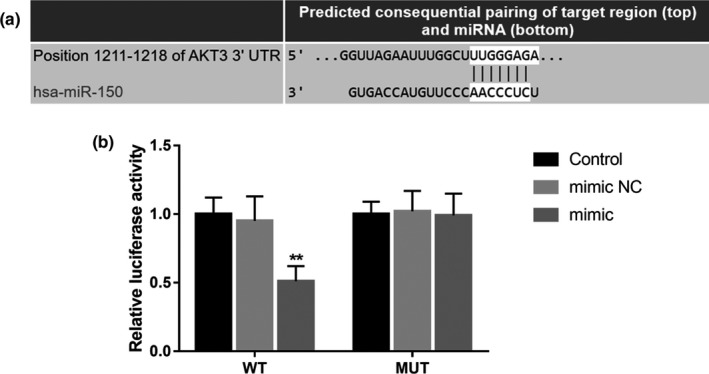
AKT3 acts as a target gene of miR‐150 in BV2 cells. (a) The complementary sequence of miR‐150 in the 3′‐UTR of AKT3. (b) The relative luciferase activity in the WT group was inhibited by the overexpression of miR‐150. **p < .01

A complementary sequence of miR‐150 was found in the 3′‐UTR of AKT3 (Figure 4a), indicating that AKT3 might be a potential target of miR‐150. The further luciferase assay shown that the relative luciferase activity of the cells transfected with WT of AKT3 was markedly suppressed by the overexpression of miR‐150 (*p* < .01, Figure 4b), whereas the cells with MUT of AKT3 had no significant change in the luciferase activity (*p* > .05).

## 4 DISCUSSION

This study was carried out to identify a novel biomarker for the diagnosis of PD and explore the regulatory effect of miR‐150 on neuroinflammation in the pathogenesis of PD. In this study, serum expression of miR‐150 in PD patients was significantly lower than that in the healthy controls, and the downregulated expression was demonstrated to serve as a candidate diagnostic biomarker of PD. Moreover, negative correlation was found between the expression of miR‐150 and the serum levels of proinflammatory cytokines. Through the in vitro experiments, this study found that the overexpression of miR‐150 in LPS‐treated BV2 cells led to the inhibited release of IL‐1β, IL‐6, and TNF‐α. The in silico prediction and luciferase assay results shown that AKT3 was a direct target of miR‐150 in BV2 cells.

Dysregulation of miRNAs has been reported to be involved in the regulation of pathogenesis in various human diseases (Singh & Sen, 2017). For example, Chen et al. found that the decreased expression of miR‐204 in hypoxic–ischemic encephalopathy tissue had regulatory effect on disease progression by regulating cell viability of neurons (Chen et al., (2019)). Liu et al. presented a study that demonstrated the decreased serum miR‐21, miR‐25, miR‐146a, and miR‐181a were potential diagnostic biomarker in autoimmune diabetes and might participate in the disease pathogenesis (Liu et al., 2019). In Alzheimer's disease, a study by Jia et al. reported that serum downregulated expression of miR‐223 in Alzheimer's disease acted as a diagnostic biomarker to distinguish patients from healthy individuals (Jia & Liu, 2016). These aforementioned studies suggested the important roles of aberrant expression of miRNAs and their clinical significance for disease diagnosis. To improve the situation of lack for noninvasive diagnostic strategies, some miRNAs with abnormal expression profiles have been identified in PD patients. A study by Ding et al. gave evidence for five miRNAs, including the elevated miR‐195 and decreased miR‐185, miR‐15b, miR‐221, and miR‐181a, as serum biomarkers for the diagnosis of PD (Ding et al., 2016). Another study by Dong et al. also reported four miRNAs (miR‐141, ‐214, ‐146b‐5p, and ‐193a‐3p) with downregulated expression as novel diagnostic biomarkers for the early diagnosis of PD (Dong et al., 2016). In this study, serum specimens were collected from PD patients and the serum expression of miR‐150 in PD patients was found to be decreased when compared with the healthy controls, indicating the potential that miR‐150 might be involved in the development of PD. Furthermore, a ROC curve constructed based on serum miR‐150 levels suggested that the downregulation of miR‐150 served as a serum biomarker for the diagnosis of PD, and a cutoff value obtained from the ROC analysis provided a reference data for serum miR‐150 level to distinguish PD patients from healthy individuals. According to the previously reported studies, the diagnostic significance of deregulated miR‐150 has been established in colorectal carcinoma (Aherne et al., 2015), acute myocardial infarction (Zhang et al., 2015), and type 1 diabetes mellitus (Wang, Gu, Xu, Zhang, & Yang, 2018). The data of our study provided a novel insight into the diagnostic value of miR‐150 in PD.

Neuroinflammation is one of the most important events during the pathogenesis of PD, and some functional miRNAs have been determined as therapeutic targets of PD through inhibiting neuroinflammatory responses. For example, miR‐7 could weaken neuroinflammation in the pathogenesis of PD by targeting Nod‐like receptor protein 3 inflammasome (Zhou et al., 2016). miR‐30e has been reported to ameliorate neuroinflammation in PD animal model and inferred as a target for PD therapy (Li et al., 2018). The close relationship between miR‐150 and inflammatory reaction has also been reported in some pathological processes (Li, Yao, Ma, & Chen, 2019). And its inhibiting effect on neuroinflammation has been reported in neuropathic pain (Ji et al., 2018). In the patients with PD, this study found that the serum miR‐150 levels were negatively correlated with the levels of proinflammatory cytokines. By using LPS‐stimulated microglia, we further verified that the overexpression of miR‐150 in BV2 cells led to the suppressed neuroinflammatory responses. Thus, there were reasons to believe that miR‐150 might be a therapeutic target for PD by ameliorating neuroinflammation.

Although the clinical significance of miR‐150 and its biological role in the inflammatory pathogenesis were analyzed in this study, the mechanisms underlying the role of miR‐150 in PD remain unclear. It is well known that AKT signaling is a star signaling pathway with pivotal roles in numerous biological processes, including the progression of PD (Jha, Jha, Kar, Ambasta, & Kumar, 2015). For example, amentoflavone exerts its protective role against dopaminergic neuron injury in PD mice by regulating the PI3K/AKT signaling pathway (Cao et al., 2017). Another study also revealed that AKT signaling mediated the effect of polydatin in LPS‐induced PD microglia model (Huang et al., 2018). Interestingly, our study found a complementary sequence of miR‐150 at the 3′‐UTR of AKT3, and demonstrated that AKT3 was a direct target of miR‐150 in BV2 cells. Thus, we deduced that the neuroinflammatory regulation of miR‐150 might be achieved by targeting AKT3. To clearly uncover the related mechanisms, further investigations are needed to confirm our results and deduction.

In conclusion, our findings might provide evidence for miR‐150 as a novel and noninvasive biomarker for PD diagnosis. The neuroinflammation in the pathogenesis of PD may be inhibited by the overexpression via targeting AKT3, suggesting that the methods to increase miR‐150 expression may be novel strategies for PD therapy.

## CONFLICTS OF INTEREST

The authors have declared no conflict of interest.
